# Expression of hypoxia-inducible factor-1α and vascular density in mammary adenomas and adenocarcinomas in bitches

**DOI:** 10.1186/1751-0147-55-73

**Published:** 2013-10-24

**Authors:** Janusz A Madej, Jan P Madej, Piotr Dziegiel, Bartosz Pula, Marcin Nowak

**Affiliations:** 1 Department of Pathology, Faculty of Veterinary Medicine, Wroclaw University of Environmental and Life Sciences, Wroclaw 50-375, Poland; 2 Department of Histology and Embryology, Wroclaw University of Environmental and Life Sciences, Wroclaw 50-375, Poland; 3 Department of Histology and Embryology, Wroclaw Medical University, Wroclaw 50-368, Poland; 4 Department of Physiotherapy, Wroclaw University School of Physical Education, Wroclaw 51-612, Poland

**Keywords:** HIF-1, Adenocarcinoma, Adenoma, Mammary gland, Dog

## Abstract

**Background:**

The study aimed at examining hypoxia-inducible factor (HIF)1α expression in adenocarcinomas and adenomas in bitches in regard to tumour malignancy grade, proliferation, apoptosis and vascularisation. Therefore, paraffin sections of 15 adenomas and 64 adenocarcinomas sampled from 79 dogs aged 6 to 16 years were analysed.

**Results:**

A significantly higher HIF-1α expression was noted in adenocarcinomas in comparison to adenomas (*P* < 0.0004). Moreover, HIF-1α expression in adenocarcinomas correlated positively with tumour malignancy grade (r = 0.59, *P* < 0.05), Ki-67 antigen expression (r = 0.43; *P* < 0.0005), TUNEL-positive cells (r = 0.62, *P* < 0001) and tumour vascularity measured by quantification of vessels characterized by the expression of von Willebrand Factor (r = 0.57, *P* < 0.05).

**Conclusion:**

Results of this study indicate a similar biological role of HIF-1α in dogs and in humans, which may confirm suitability of the animal model in investigations on progression of tumours in humans.

## Background

Neoplastic cells and cells of malignant tumours at their preliminary stage of development are supplied, in particular, with oxygen and metabolic products *via* diffusion. This secures conditions for their growth and attainment of a tumour diameter not around 2 mm. Subsequent growth of the tumour exceeding this size requires additional supply provided by blood vessels. Therefore, hypoxia develops within neoplastic tissue and tumour cells begin to manifest an increased demand for glucose and an accelerated glycolysis, in such conditions securing the principal source of ATP [1]. Glycolysis was shown to progress very efficiently in tumours growing in hypoxic conditions since they express hypoxia-inducible factor (HIF-1). This factor is responsible for an increased expression of several proteins, including glycolytic enzymes such as hexokinase-1 and -3, phosphofructokinase L, aldolase A and C, phosphoglycerate kinase-1, enolase-1, lactate dehydrogenase and the so called glucose transporters GLUT-1 and GLUT-3 [1]. Intensity of glucose uptake by tumour cells was found to manifest positive correlation with their aggressiveness [1]. Moreover, HIF-1 stimulates tumour growth by activation of the *VEGF* gene transcription, which codes for vascular endothelial growth factor, the principal inducer of angiogenesis. In the absence of neovascularization tumour growth would be inhibited or even the tumour would show regression. Anti-neoplastic therapy takes advantage of this phenomenon by inhibiting angiogenesis in a tumour e.g. using monoclonal VEGF-specific antibodies [1,2]. It should be added that HIF-1 stimulates also transcription of *IGF2* gene, coding for insulin-like growth factor 2 (IGF2), which facilitates survival of tumour cells also in an environment with an diminished oxygen content [1,3].

HIF-1 is a heterodimer, consisting of HIF-1α and HIF-1β subunits. The HIF-1β subunit undergoes a constitutive expression while the expression of HIF-1α is low in most cells in normoxia conditions. Inhibition of HIF-1α expression results from activity of oxygen-dependent hydroxylases which enzymatically modify HIF-1α chain enabling its binding with von Hippel-Lindau tumour suppressor protein (VHL) [4]. In turn, VHL acts as a recognition factor for ubiquitin-protein ligase E3, which directs HIF-1α to degradation in proteasomes [5,6]. In normoxia conditions, half-life of HIF-1α protein is very short but it becomes markedly elongated in hypoxia [7]. Stimulation of HIF-1α synthesis utilizes the signalling pathway leading to a tyrosine kinase receptor, such as HER2 (Human Epidermal Growth Factor Receptor 2), with mediation of phosphatidylinositol-3-kinase (PI3K), serine/threonine kinases (AKT) and mammalian target for rapamycin (mTOR) [8]. The signalling pathway is inhibited by PTEN protein (phosphatase and tensin homologue deleted on chromosome ten), which dephosphorylates the product of PI3K reaction [1]. Therefore, HIF-1 may be regarded as a factor, which allows the cells to adapt to low tissue levels of oxygen.

Our study aimed at demonstration of HIF-1α protein expression and determination of its intensity in the most frequently manifested malignant and benign mammary tumours of epithelial origin (adenocarcinomas and adenomas) in bitches. Moreover, an attempt was made to correlate the obtained results with expression levels of the Ki-67 proliferation antigen and with blood vessel density of the tumours.

## Methods

The research we performed was approved and financed by the National Science Center of Poland. As this research was performed on archival material routinely collected during surgical-treatment procedures and no additional harm was done to the animals due to the experiments, we did not require an additional ethics approval for our research. All the experiments were performed on disposable material which were not utilized for future scientific experiments. Only paraffin-embedded tissues were used for the study.

### Tissue material and immunohistochemistry (IHC)

Material for the study was sampled during surgery in 79 female dogs of various breeds, aged 6 to 16 years. The tumours were verified by histopathological examination of the HE sections and represented adenomas (15 cases) and adenocarcinomas (64 cases).

Formalin-fixed, paraffin-embedded tissue was freshly cut (4 μm). The sections were mounted on Superfrost Plus slides (Menzel Gläser, Braunschweig Germany) and subsequently deparaffinised by boiling in Antigen Retrieval Solution (High pH = 9 for HIF-1α, Low pH = 6 for Ki-67; DakoCytomation, Glostrup, Denmark) using PT Link Rinse Station (DakoCytomation). Then, the sections were incubated (20 min; room temperature, RT) in Link48 automated staining platform (DakoCytomation) utilizing murine primary monoclonal antibodies diluted in the Background Reducing Antibody Diluent (DakoCytomation) and directed against HIF-1α (1:600; Novus Biologicals, Littleton, USA), von Willbrand Factor (vWF; 1:800; DakoCytomation) or Ki-67 (ready-to-use, DakoCytomation). The visualization of the studied antigens was performed using EnVision FLEX (DakoCytomation), according to the manufacturer’s instructions. All the sections were counterstained with Meyer’s hematoxylin. In all the cases, controls were included, in which specific antibody was substituted by the Primary Negative Control (DakoCytomation).

Apoptosis detection was performed utilizing the ApopTag® Peroxidase In Situ Apoptosis Detection Kit (Millipore, Billerica, USA). Paraffin sections were dewaxed in xylene, rehydrated in alcohol and rinsed in distilled water and 1xPBS, pH 7.4. Then, the sections were incubated in Proteinase K (DakoCytomation) for 5 min in RT and rinsed in 1xPBS. Endogenous peroxidase was blocked by 5 min incubation in 3% H_2_O_2_/1xPBS. Subsequently, the sections were incubated with Equilibration Buffer for 10 min in RT, with subsequent incubation with TdT Enzyme and Reaction Buffer at 37°C for 1 h. The reaction was stopped after 10 min incubation in the Stop Buffer and rinsed in 1xPBS. Then, anti-dioxygenin peroxidase-conjugated antibodies were applied for 30 min at RT. Following that, the sections were incubated for 10 min with diaminobenzidine (DAB; DakoCytomation) to visualize the TUNEL-positive cell nuclei. Finally, the sections were counterstained with Mayer’s hematoxylin and, after dehydration in alcohols, mounted in SUB-X Mounting Medium (both DakoCytomation).

### Quantification of IHC reactions

Microphotographs of all the studied tumours were subjected to computer-assisted image analysis *via* a computer coupled to an Olympus BX53 optical microscope (Olympus, Japan). The set had the potential to record images and to perform their digital analysis. The measurements took advantage of Cell^A^ software (Olympus Soft Imaging Solution GmbH, Germany).

Microscope examination allowed determination of the malignancy grade of the adenocarcinomas. The grade was established using the scale of Bloom-Richardson in modification of Elston and Ellis [9]. The evaluation method of the malignancy grade included three parameters scored in the scale from 0 to 3 points: formation of tubules (evident, moderate, slight), polymorphism of cell nuclei (slight, moderate, marked), number of mitotic figures per 10 microscope fields at the magnification of × 400 (0–7, 8–16, ≥ 17). The sum of the points provided potential to distinguish three malignancy grades (G) among the tumours: 0–5 pts. – G1, 6–7 pts. – G2, 8–9 pts. – G3.

Expression of HIF-1α was appraised using the modified semi-quantitative immunoreactive score (IRS) scale according to Remmele (Table 1) [10]. The method takes into account both proportion of positively stained cells and intensity of the colour reaction, while the final score is the product of the parameters, with values ranging from 0 to 12 points (no reaction = 0 points (-); weak reaction = 1–2 points (+), moderate reaction = 3–4 points (++), intense reaction = 6–12 points (+++)).

**Table 1:** Semi-quantitative immunoreactive score (IRS) taking into account both the percentage of stained cells (A) and the intensity of reaction product (B) in which the final results correspond to the product of the two variables (AxB)

**Point score**	**A**	**B**
0	No cells with positive reaction	No colour reaction
1	≤ 10% Cells with positive reaction	Low intensity of colour reaction
2	11-50% Cells with positive reaction	Average intensity of colour reaction
3	51-80% Cells with positive reaction	Intense colour reaction
4	> 80% Cells with positive reaction	

Microvessel density (MVD) of vWF-positive vessels was quantified under × 200 magnification in five intratumoural areas of the lesion and the final score was determined as a mean of the five quantified areas.

The Ki-67 antigen expression and TUNEL stained sections were scored under × 400 magnification in five areas, in which number of positive cells presenting brown reaction colour were counted. The final score represented the percentage of positive tumour cells to all tumour cells in the examined sections.

### Statistical analysis

The results were subjected to statistical analysis using Prism 5.0 (GraphPad, La Jolla, USA) software, employing Mann–Whitney test and Spearman’s correlation analysis. In all the analyses, results were considered statistically significant when *P* < 0.05.

## Results

**Figure 1:**
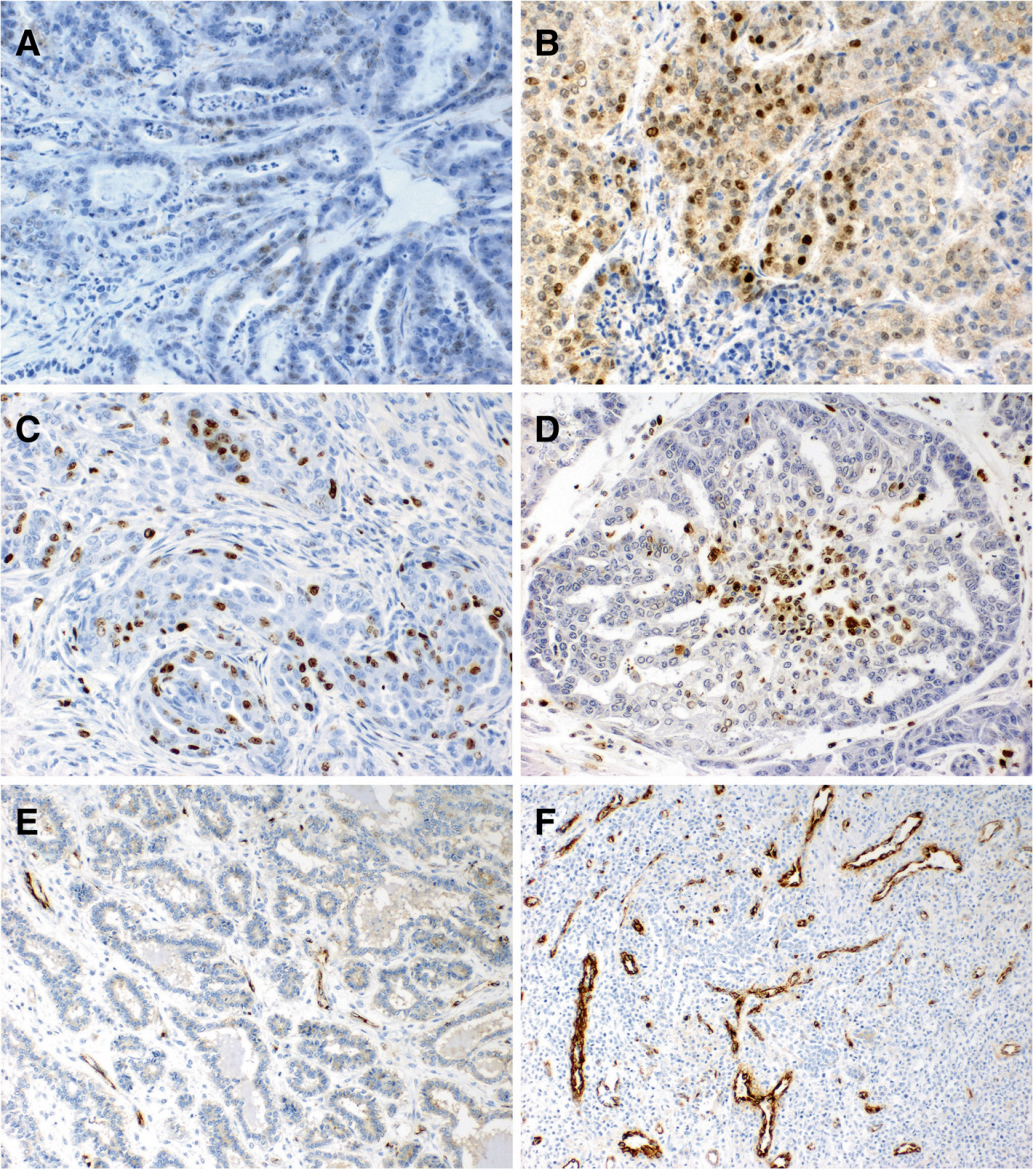
**Immunohistochemical expression of HIF-1α in adenoma (A) and adenocarcinoma (B).** High expression intensity of Ki-67 antigen **(C)** and TUNEL in cancer cells **(D)**. Von Willbrand Factor (vWF) expression in endothelial cells noted in adenoma **(E)** and adenocarcinoma **(F)**. Magnification × 200 **(A**-**D)**; ×40 **(E**-**F)**.

**Figure 2:**
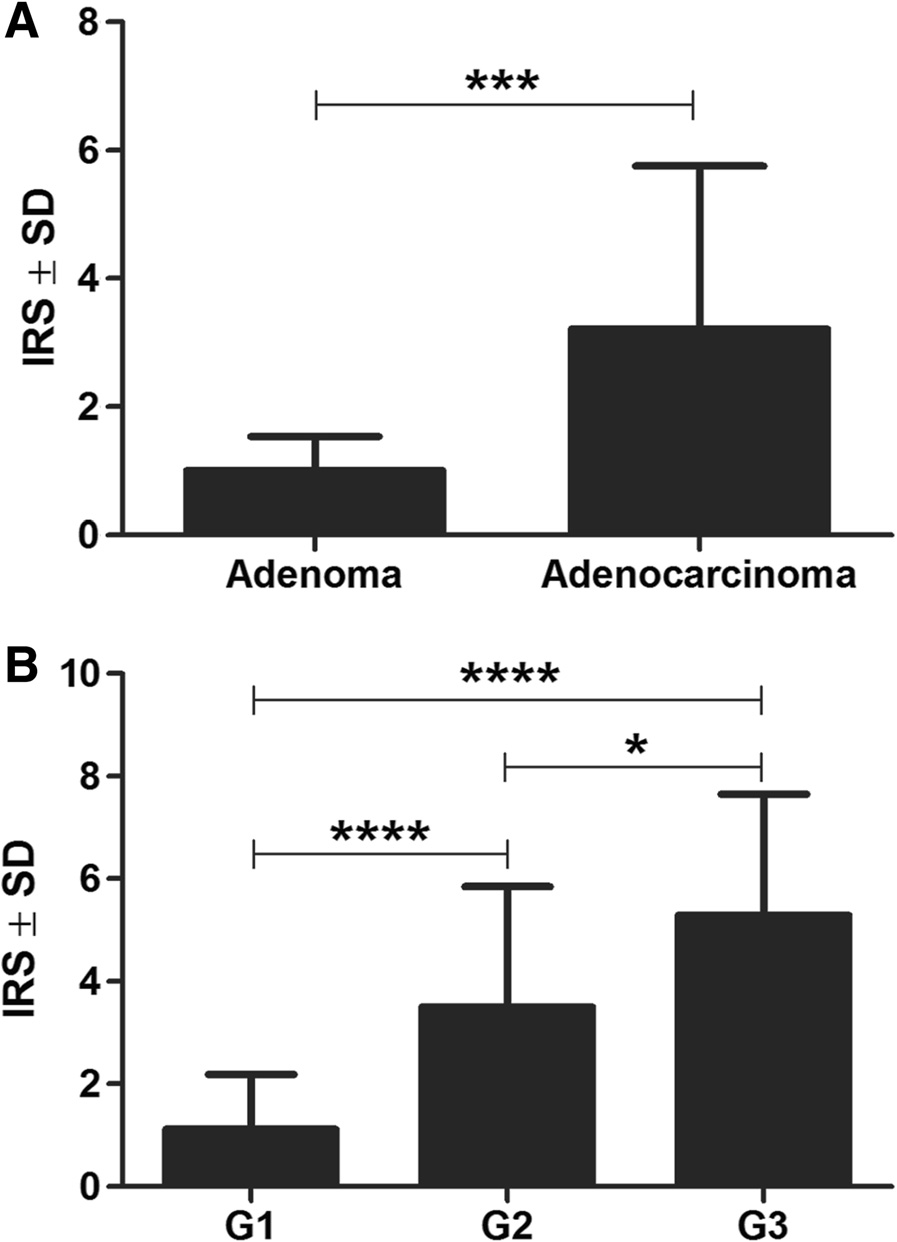
**Hypoxia-inducible factor-1α (HIF-1α) expression in adenomas and adenocarcinomas (A) and in relation to the grade of malignancy of the studied adenocarcinomas (B).** ***P < 0.001; ****P < 0.0001, Mann–Whitney test.

Expression of HIF-1α protein was demonstrated both in mammary adenomas and adenocarcinomas in bitches (Figures 1A, 1B). In all the cases with positive reaction (90% adenocarcinomas and in 86% adenomas) a nuclear-cytoplasmic HIF-1α expression was noted. Moreover, in the two groups of tumours evident differences were seen in intensity of the protein expression. In adenocarcinomas, over 39% tumours manifested HIF-1α expression evaluated at +, over 26% expression evaluated at ++ and 25% at +++. In adenomas, 86% of the examined tumours manifested expression of the protein which, however, did not exceed the +intensity. It should be noted that over 9% of adenocarcinomas and over 13% of adenomas manifested no HIF-1α expression. Statistical analysis using Mann-Whitney test demonstrated a significantly higher HIF-1α expression in adenocarcinomas than in adenomas (*P* = 0.0004) (Figure 2A).

The relationship between distribution of HIF-1α expression intensity and malignancy grade was also of interest. In G1 adenocarcinomas almost 28% of the tumours manifested no HIF-1α expression, 61% of them manifested the expression at + level and 11% at ++ level. In cases of G2 adenocarcinomas, 3% of the tumours manifested no HIF-1α expression, over 37% showed + expression, over 37% of them demonstrated ++ expression and almost 22% +++ expression. G3 adenocarcinomas exhibited HIF-1α expression at + level in over 14% of cases, at ++ level in over 28% of cases and at +++ level in over 57% of cases. It should be added that only in the group of G3 adenocarcinomas over 50% of the tumours manifested high expression of the protein (+++) while no such strong HIF-1α expression could be noted in G1 or G2 tumours.

Spearman’s correlation test demonstrated a pronounced positive correlation between HIF-1α expression and tumour malignancy grade (r = 0.59; *P* < 0.05). Mann–Whitney test revealed significant differences in HIF-1α expression between particular malignancy grades of adenocarcinomas (G1 *vs*. G2 - *P* < 0.0001; G1 *vs*. G3 - *P* < 0.0001; G2 *vs*. G3 – *P* = 0.02) (Figure 2B).

**Figure 3:**
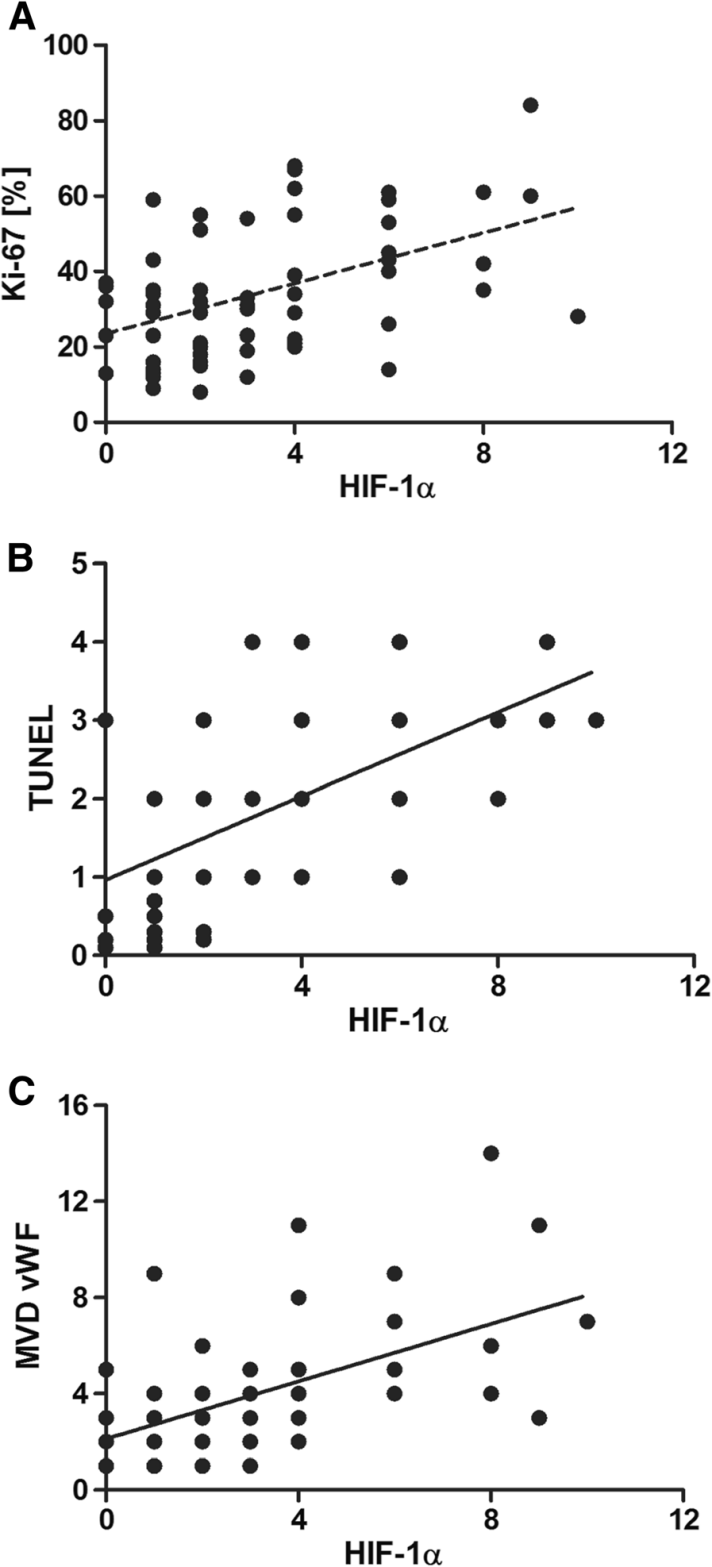
Correlations between expressions of HIF-1α, Ki-67 antigen (A), TUNEL (B) and vWF MVD (C) in the analyzed adenocarcinomas.

Tumour cell proliferation was determined by assessing the expression of the Ki-67 antigen (Figure 1C). Similarly, positive correlations were disclosed between tumour’s malignancy grade and expression of Ki-67 proliferation antigen (r = 0.61; *P* < 0.0001), and between expressions of HIF-1α and Ki-67 (r = 0.43; *P* < 0.0005; Figure 3A).

Studies on intensity of apoptosis using the TUNEL approach (Figure 1D) demonstrated that level of apoptosis in canine mammary adenocarcinomas manifested positive correlation with expression of HIF-1α protein and the correlation demonstrated a high level (r = 0.62; *P* < 0.0001; Figure 3B).

**Figure 4:**
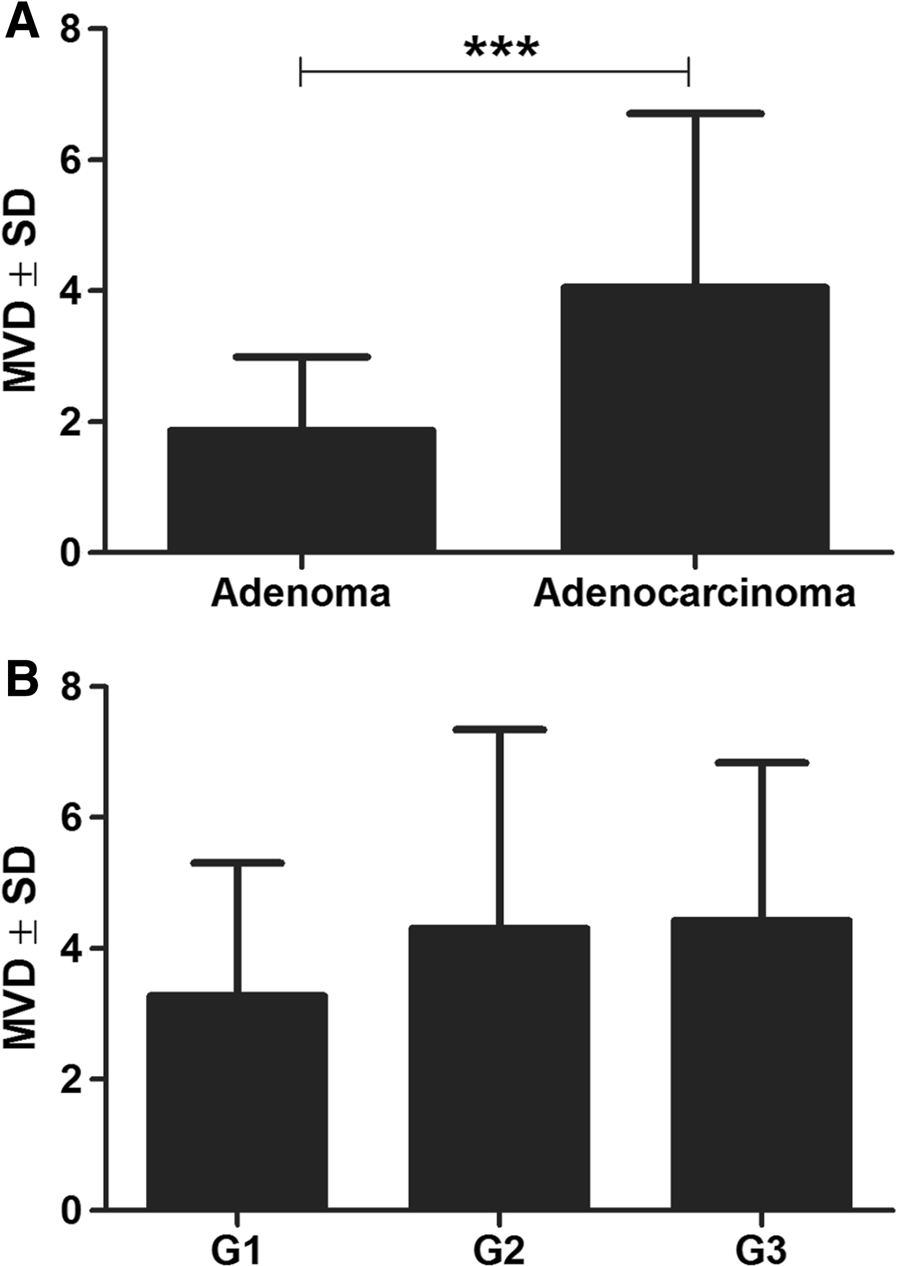
**Von Willbrand Factor mean vascular density (vWF MVD) in adenomas and adenocarcinomas (A) and in relation to the grade of malignancy of the studied adenocarcinomas (B).** ***P < 0.001, Mann–Whitney test.

An extremely important and interesting aspect of the study was the examination of a correlation between HIF-1α protein expression and vWF MVD (Figures 1E, 1F). Both in adenomas and in adenocarcinomas the correlation proved to be pronounced and positive. Its level was slightly higher in malignant tumours (adenocarcinomas: r = 0.57; *P* < 0.05; Figure 3C) than in benign tumours (adenomas r = 0.52; *P* < 0.05). Mann–Whitney test demonstrated significant differences in vWF MVD between individual malignancy grades (G1 *vs*. G2 – *P* = 0.251; G1 *vs*. G3 – *P* = 0.098; G2 *vs*. G3 – *P* = 0.586) – Figure 4.

## Discussion

Immunohistochemical studies on human tumours demonstrated pronounced expression of HIF-1α in many common tumours, which probably represents a consequence of both hypoxia within the tumour and various genetic disturbances in neoplastic cells [11]. Moreover, HIF-1α expression was shown to manifest positive correlation with expression of VEGF and with density of microvessels in most of tumours of central nervous system [12], ovarian cancer [13], ductal mammary carcinoma [14], colon adenocarcinoma [15], endometrial adenocarcinoma [16], ductal pancreatic adenocarcinoma [17], small-cell pulmonary carcinoma [18]. The relationship seems to be of significance since not only elevated HIF-1α expression is linked to increased VEGF levels, but absence of HIF-1α expression in several cases results in a decreased VEGF level and, thus, an inhibited process of neoangiogenesis. The latter observation has been corroborated in studies on embryonal stem cells of mice devoid of HIF-1α expression, in which VEGF mRNA level was markedly decreased and could not be induced by hypoxia. Similarly in our study we have demonstrated a pronounced positive correlation between expression of HIF-1α and density of microvessels both in a malignant (adenocarcinoma) and benign (adenoma) tumours, even if in the latter case the correlation has shown a slightly lower level. However, it should be added that some reports documented absence of a significant correlation between expressions of mRNA for HIF-1α and VEGF in hypophyseal adenomas [19]. Their authors suggested that in such tumours VEGF expression may exhibit no pronounced dependence on expression of HIF-1α.

A very important element of HIF-1α biological activity is the induction of protein synthesis involved, i.a., in development of metastases, including vimentin, fibronectin, metalloproteinase 2, cathepsin D, urokinase-type plasminogen activator receptor (uPAR). Moreover, HIF-1α protein can induce a decrease in E-cadherin expression, the protein important for cell adhesion [20]. In studies on *in vitro* invasiveness of human pulmonary adenocarcinoma cells, Shyu et al. [21] demonstrated that in conditions of normoxia, HIF-1α manifested expression in cells presenting high invasiveness (CL1-5) while no expression of the factor was noted in cells of a low invasiveness (CL1). In parallel, the authors found that an increased HIF-1α expression in cells of human pulmonary carcinoma was linked to their increased invasiveness, probably due to an increased expression of uPAR receptor and metalloproteinase 1 and 2 (MMP1 and MMP2). In our study we have analogously found that HIF-1α expression has manifested positive correlation with certain prognostically unfavourable traits of the tumours, i.e., with malignancy grade and proliferative potential, reflected in expression of the Ki-67 antigen.

Shin et al. [18] found that levels of HIF-1α and VEGF mRNA expression were higher in tissues of an early or advanced colon carcinoma than in colon adenoma. They suggested that MMP-2, HIF-1α and VEGF might represent useful parameters for detection of early carcinogenesis and progression of colon carcinoma. In turn, Osada et al. [13] showed that HIF-1α expression in cell nucleus and HIF-2α in cytoplasm of neoplastic cells was linked to an unfavourable prognosis in women with ovarian carcinoma. The authors found also that HIF-1α expression in cell nucleus represented an independent prognostic factor in women with such a cancer. Similar results were obtained by Daponte et al. [22], who noted that survival of females with serous ovarian carcinoma, the cells of which demonstrated strong HIF-1α expression, was significantly abbreviated as compared to patients in whom neoplastic cells showed low or no HIF-1α expression. A pronounced expression of HIF-1α is linked to an increased risk of death at early stages of various types of malignant tumours, including carcinomas of uterine cervix, esophageal, mammary carcinoma and cerebral oligodendroglioma [12,23-25]. Moreover, reports are available which show that, augmented expression of HIF-1α correlated with a increased apoptosis of neoplastic cells [25,26]. Similarly, in our investigations on canine mammary adenocarcinomas we have found that an increased HIF-1α expression was paralleled by higher levels of apoptosis among neoplastic cells. This may indicate that certain tumour cell subpopulations are more sensitive to an increasingly intense hypoxia. Moreover, it has been demonstrated that experimentally induced overexpression of HIF-1α in cells of non-small-cell pulmonary carcinoma (line A549) inhibits tumour development, i.a., through a more pronounced apoptosis [3].

## Conclusions

High expression of HIF-1α in neoplastic cells, expressing their adjustment to hypoxia conditions in the tumour, in the course of why some tumours may provide a useful marker of canine tumour aggressiveness based on observed positive correlations with the tumour malignancy grade and the Ki-67 antigen expression. The HIF-1α expression in mammary adenomas and adenocarcinomas of bitches may indicate that the biological role of the protein is similar in tumours of canines and humans. This may confirm suitability of using the animal model in studies on progression of tumours in the man.

## Competing interests

The authors declare that they have no competing interests.

## Authors’ contributions

MN and JAM initiated and planned the study, MN, JPM and PD completed tissue processing, staining and performed the IHC, JPM and BP performed the statistical analysis, compiled the results and drafted the manuscript. All authors were significantly involved in designing the study, interpreting of data and composing the manuscript. All authors read and approved the final manuscript.

## References

[B1] Semenza GL HIF-1 and tumor progression: pathophysiology and therapeutics Trends Mol Med 2002 55 62 67 10.1016/S1471-4914(02)02279-7 11927290

[B2] Braghiroli MI Sabbaga J Hoff M Bevacizumab: overview of the literature Expert Rev Anticancer Ther 2012 55 567 580 10.1586/era.12.13 22594892

[B3] Savai R Schermuly RT Voswinckel R Renigunta A Reichmann B Eul B Grimminger F Seeger W Rose F Hänze J HIF-1alpha attenuates tumor growth in spite of augmented vascularization in an A549 adenocarcinoma mouse model Int J Oncol 2005 55 393 400 16010420

[B4] Epstein AC Gleadle JM McNeill LA Hewitson KS O’Rourke J Mole DR Mukherji M Metzen E Wilson MI Dhanda A Tian YM Masson N Hamilton DL Jaakkola P Barstead R Hodgkin J Maxwell PH Pugh CW Schofield CJ Ratcliffe PJ *C. elegans* EGL-9 and mammalian homologs define a family of dioxygenases that regulate HIF by prolyl hydroxylation Cell 2001 55 43 54 10.1016/S0092-8674(01)00507-4 11595184

[B5] Ivan M Kondo K Yang H Kim W Valiando J Ohh M Salic A Asara JM Lane WS Kaelin WG Jr HIFα targeted for VHL-mediated destruction by proline hydroxylation: implications for O2 sensing Science 2001 55 464 468 10.1126/science.1059817 11292862

[B6] Jaakkola P Mole DR Tian YM Wilson MI Gielbert J Gaskell SJ Kriegsheim A Hebestreit HF Mukherji M Schofield CJ Maxwell PH Pugh CW Ratcliffe PJ Targeting of HIF-α to the von Hippel–Lindau ubiquitylation complex by O2-regulated prolyl hydroxylation Science 2001 55 468 472 10.1126/science.1059796 11292861

[B7] Huang LE Arany Z Livingston DM Bunn HF Activation of hypoxia-inducible transcription factor depends primarily upon redox-sensitive stabilization of its a subunit J Biol Chem 1996 55 32253 32259 10.1074/jbc.271.50.32253 8943284

[B8] Laughner E Taghavi P Chiles K Mahon PC Semenza GL HER2 (neu) signaling increases the rate of hypoxia-inducible factor 1α (HIF-1 α) synthesis: novel mechanism for HIF-1-mediated vascular endothelial growth factor expression Mol Cell Biol 2001 55 3995 4004 10.1128/MCB.21.12.3995-4004.2001 11359907 PMC87062

[B9] Elston CW Ellis IO Pathological prognostic factors in breast cancer: experience from a large study with long-term follow-up Histopathology 1991 55 403 410 10.1111/j.1365-2559.1991.tb00229.x 1757079

[B10] Remmele W Stegner HE Vorschlag zur einheitlichen Definition eines immunoreaktiven Score (IRS) fur den immunohistochemichen Ostrogenrezeptor-Nachweis (ER-ICA) im Mammakarzinomgewebe Pathologie 1987 55 138 140 3303008

[B11] Talks KL Turley H Gatter KC Maxwell PH Pugh CW Ratcliffe PJ Harris AL The expression and distribution of the hypoxia-inducible transcription factors HIF-1α and HIF-2α in normal human tissues, cancers, and tumor-associated macrophages Am J Pathol 2000 55 411 421 10.1016/S0002-9440(10)64554-3 10934146 PMC1850121

[B12] Birner P Gatterbauer B Oberhuber G Schindl M Rössler K Prodinger A Budka H Hainfellner JA Expression of hypoxia-inducible factor-1α in oligodendrogliomas: its impact on prognosis and on neoangiogenesis Cancer 2001 55 165 171 10.1002/1097-0142(20010701)92:1<165::AID-CNCR1305>3.0.CO;2-F 11443623

[B13] Osada R Horiuchi A Kikuchi N Yoshida J Hayashi A Ota M Katsuyama Y Mellilo G Konishi I Expression of hypoxia-inducible factor 1α, hypoxia-inducible factor 2α, and von Hippel–Lindau protein in epithelial ovarian neoplasms and allelic loss of von Hippel-Lindau gene: nuclear expression of hypoxia-inducible factor 1α is an independent prognostic factor in ovarian carcinoma Hum Pathol 2007 55 1310 1320 10.1016/j.humpath.2007.02.010 17555795

[B14] Bos R Zhong H Hanrahan CF Mommers EC Semenza GL Pinedo HM Abeloff MD Simons JW van Diest PJ van der Wall E Levels of hypoxia-inducible factor-1α during breast carcinogenesis J Natl Cancer Inst 2001 55 309 314 10.1093/jnci/93.4.309 11181778

[B15] Jiang CQ Fan LF Liu ZS Qian Q Xia D Diao LM He YM Ai ZL Expression levels and significance of hypoxia inducible factor-1 alpha and vascular endothelial growth factor in human colorectal adenocarcinoma Chin Med J 2004 55 1541 1546 15498380

[B16] Ozbudak IH Karaveli S Simsek T Erdogan G Pestereli E Neoangiogenesis and expression of hypoxia-inducible factor 1alpha, vascular endothelial growth factor, and glucose transporter-1 in endometrioid type endometrium adenocarcinomas Gynecol Oncol 2008 55 603 608 10.1016/j.ygyno.2007.11.028 18191183

[B17] Sun HC Qiu ZJ Liu J Sun J Jiang T Huang KJ Yao M Huang C Expression of hypoxia-inducible factor-1 alpha and associated proteins in pancreatic ductal adenocarcinoma and their impact on prognosis Int J Oncol 2007 55 1359 1367 17487356

[B18] Shin JE Jung SA Kim SE Joo YH Shim KN Kim TH Yoo K Moon IH Expression of MMP-2, HIF-1alpha and VEGF in colon adenoma and colon cancer Korean J Gastroenterol 2007 55 9 18 18172354

[B19] Kim K Yoshida D Teramoto A Expression of hypoxia-inducible factor 1alpha and vascular endothelial growth factor in pituitary adenomas Endocr Pathol 2005 55 115 121 10.1385/EP:16:2:115 16199896

[B20] Brahimi-Horn MC Pouysségur J Harnessing the hypoxia-inducible factor in cancer and ischemic disease Biochem Pharmacol 2007 55 450 457 10.1016/j.bcp.2006.10.013 17101119

[B21] Shyu KG Hsu FL Wang MJ Wang BW Lin S Hypoxia-inducible factor 1alpha regulates lung adenocarcinoma cell invasion Exp Cell Res 2007 55 1181 1191 10.1016/j.yexcr.2007.01.013 17335808

[B22] Daponte A Ioannou M Mylonis I Simos G Minas M Messinis IE Koukoulis G Prognostic significance of hypoxia-inducible factor 1 alpha(HIF-1alpha) expression in serous ovarian cancer: an immunohistochemical study BMC Cancer 2008 55 1 10 10.1186/1471-2407-8-335 PMC2651893 19014607

[B23] Birner P Schindl M Obermair A Plank C Breitenecker G Oberhuber G Overexpression of hypoxia-inducible factor 1α is a marker for an unfavorable prognosis in early-stage invasive cervical cancer Cancer Res 2000 55 4596 4693 10987269

[B24] Ogawa K Chiba I Morioka T Shimoji H Tamaki W Takamatsu R Nishimaki T Yoshimi N Murayama S Clinical significance of HIF-1alpha expression in patients with esophageal cancer treated with concurrent chemo radiotherapy Anticancer Res 2011 55 2351 2359 21737664

[B25] Koukourakis MI Giatromanolaki A Skarlatos J Corti L Blandamura S Piazza M Gatter KC Harris AL Hypoxia inducible factor (HIF-1a and HIF-2a) expression in early esophageal cancer and response to photodynamic therapy and radiotherapy Cancer Res 2001 55 1830 1832 11280732

[B26] Zhong H De Marzo AM Laughner E Lim M Hilton DA Zagzag D Buechler P Isaacs WB Semenza GL Simons JW Overexpression of hypoxia-inducible factor 1a in common human cancers and their metastases Cancer Res 1999 55 5830 5835 10582706

